# BIRC3 is a biomarker of mesenchymal habitat of glioblastoma, and a mediator of survival adaptation in hypoxia-driven glioblastoma habitats

**DOI:** 10.1038/s41598-017-09503-8

**Published:** 2017-08-24

**Authors:** Dapeng Wang, Anders E. Berglund, Rajappa S. Kenchappa, Robert J. MacAulay, James J. Mulé, Arnold B. Etame

**Affiliations:** 1 Department of Neuro-Oncology, H. Lee Moffitt Cancer Center and Research Institute, 12902 Magnolia Drive, Tampa, FL 33612 USA; 2 Department of Biostatistics and Bioinformatics, H. Lee Moffitt Cancer Center and Research Institute, 12902 Magnolia Drive, Tampa, FL 33612 USA; 3 Department of Anatomic Pathology, and H. Lee Moffitt Cancer Center and Research Institute, 12902 Magnolia Drive, Tampa, FL 33612 USA; 4 Department of Immunology, H. Lee Moffitt Cancer Center and Research Institute, 12902 Magnolia Drive, Tampa, FL 33612 USA; 5 Mayo Clinic, Jacksonville, FL 32224 USA

## Abstract

Tumor hypoxia is an established facilitator of survival adaptation and mesenchymal transformation in glioblastoma (GBM). The underlying mechanisms that direct hypoxia-mediated survival in GBM habitats are unclear. We previously identified BIRC3 as a mediator of therapeutic resistance in GBM to standard temozolomide (TMZ) chemotherapy and radiotherapy (RT). Here we report that BIRC3 is a biomarker of the hypoxia-mediated adaptive mesenchymal phenotype of GBM. Specifically, in the TCGA dataset elevated *BIRC3* gene expression was identified as a superior and selective biomarker of mesenchymal GBM versus neural, proneural and classical subtypes. Further, BIRC3 protein was highly expressed in the tumor cell niches compared to the perivascular niche across multiple regions in GBM patient tissue microarrays. Tumor hypoxia was found to mechanistically induce BIRC3 expression through HIF1-alpha signaling in GBM cells. Moreover, in human GBM xenografts robust BIRC3 expression was noted within hypoxic regions of the tumor. Importantly, selective inhibition of BIRC3 reversed therapeutic resistance of GBM cells to RT in hypoxic microenvironments through enhanced activation of caspases. Collectively, we have uncovered a novel role for BIRC3 as a targetable biomarker and mediator of hypoxia-driven habitats in GBM.

## Introduction

Despite aggressive therapy glioblastoma multiforme (GBM) is a highly resistant and lethal primary brain tumor with a median survival of only 14 months^1^. Treatment failures in GBM are associated with tumor-specific factors, the tumor microenvironment or a combination thereof. For example, as GBM cells rapidly proliferate they surpass the capacity of tumor vessels to adequately perfuse the tumor, resulting in hypoxia and adaptations that lead to therapy resistance^2–9^.

Recent advances in classification have revealed *IDH1/IDH2* mutations as a defining feature of the majority of adult low grade gliomas, and of a subset of adult GBMs^10^. In addition, expression analyses of The Cancer Genome Atlas (TCGA)^11^ data recently segregated GBMs into four subtypes – neural, proneural, classical and mesenchymal GBM^10^. The mesenchymal subtype is usually IDH-wildtype, and is the most aggressive and therapeutically resistant phenotype^10^.

**Figure 1:**
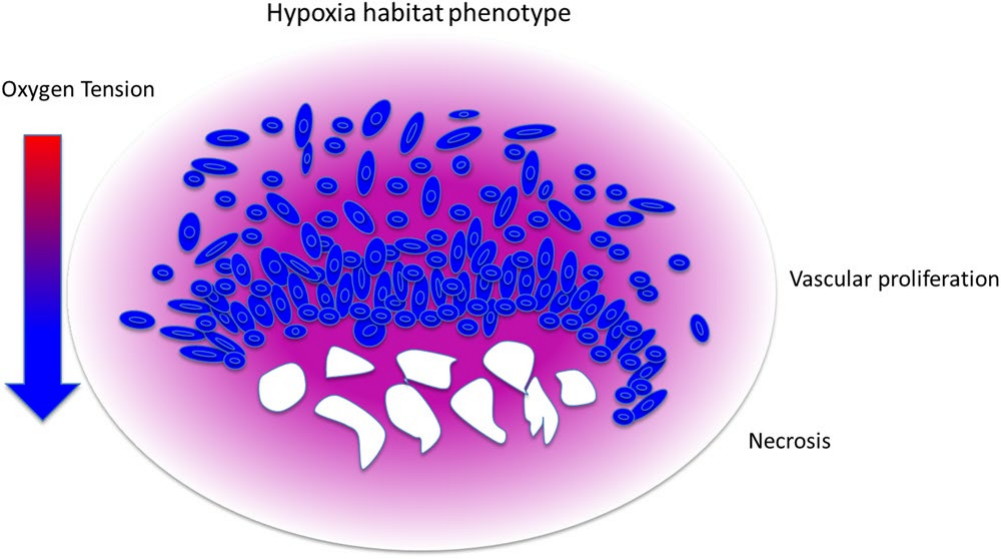
Hypoxia-Adaptive Habitat in GBM. Schematic of the hypoxia-adaptive habitat in GBM with phenotypic change: necrotic, vascular proliferation and mesenchymal transformation. The hypoxia gradient is believed to be the selective pressure and the survival adaptation within these habitat.

There is mounting evidence that regional hypoxia drives mesenchymal transformation and therapy resistance in GBM^12–14^. The pathognomonic pseudopalisading necrosis in GBM is manifest as the presence of increased cell density adjacent to areas of necrosis that are driven by hypoxia^15, 16^. These necrosis-adjacent hypercellular GBM habitats are highly hypoxic due to inadequate delivery of blood supply and nutrients (Fig. 1). Accordingly, gene expression analysis revealed the expression of hypoxia signature genes in GBM that independently correlate with aggressive tumor behavior^17–19^. Finally, clinical data indicate that the extent of necrosis (hypoxia) in GBM connotes aggressive disease and dismal survival^14, 20^.

Hypoxia-inducible factor (HIF) family genes (HIF-1α and HIF-2α) are critical regulators of the hypoxic response^21, 22^. These transcription factors are stabilized following exposure of cells to hypoxia and bind to hypoxia-response elements (HRE) found in their targets genes as heterodimers with ARNT protein, resulting in activation of several hypoxia adaptation genes that play roles in angiogenesis, invasion and cell survival^23, 24^. Hypoxia-mediated tumor invasion, and angiogenesis are well established in GBM^25, 26^, but how hypoxia promotes survival of GBM cells in reponse to therapy is poorly understood. Autophagy has been postulated as a potential mechanism of hypoxia-mediated survival adapatation in cancer cells^27–29^, yet its role in GBM remains controversial^30^.

We recently identified the anti-apoptotic protein BIRC3 as a mediator of therapeutic resistance and as a prognostic marker of temozolomide (TMZ) and radiotherapy (RT) response in GBM^31^. In the same study, we observed an increase in BIRC3 expression with the acquisition of TMZ and RT resistance. We further showed that STAT3 and PI3K signaling axes were drivers of BIRC3 up-regulation in GBM, and that that BIRC3 was required for survival adaption in GBM in response to TMZ and RT treatments. Interestingly, BIRC3 was previously reported as part of a signature gene-cluster associated with hypoxia-related inflammation in GBM^32^. However, BIRC3 has never been mechanistically implicated in hypoxia-mediated survival adaptation in GBM.

Given our previous findings with BIRC3 in GBM^31^, we hypothesized that BIRC3 might be a putative mediator and biomarker of hypoxia-driven survival adaptation in mesenchymal GBM, whereby hypoxia was a potential driver of BIRC3 expression. In support of this hypothesis, here we report that BIRC3 expression discretely segregates hypoxic mesenchymal GBM habitats from the other GBM subtype habitats. Importantly, we show mechanistically that hypoxia drives BIRC3 expression in human GBM cells through HIF-1α signaling. Lastly we show that BIRC3 is necessary for hypoxia-mediated survival adaptation in GBM through suppression of caspases. Hence BIRC3 is a targetable biomarker of hypoxia-driven habitats in GBM.

## Materials and Methods

### Mice

6–8 weeks female NCRNU athymic mice were obtained from Taconic Biosciences. All animals were housed in the AALAC–accredited Animal Resource Center of the Moffitt Cancer Center. All animal studies were performed under protocols approved by the Institutional Animal Care and Use Committee.

### The Cancer Genome Atlas (TCGA) analysis of BIRC3 expression in GBM subtypes

The Affymetrix U133A CEL files (n = 548) were normalized with IRON^33^ using sample 5500024037497121008340.A12.CEL as median sample. The CEL file with the smallest RMSD was selected if there were multiple CEL files for a single sample, resulting in 529 files. The molecular subtypes for these GBM samples were as described^10^, resulting in 53 mesenchymal, 25 neural, 52 proneural and 34 classical GBM samples. Group comparisons were performed using Mann-Whitney test and the two-tailed p-value is reported. Fold change is reported as fold change (fc). Box plots and scatter plots were generated using MATLAB R2016a (TheMathWorks, Natick, MA, USA) and the “Alternative box plot” toolbox and “gramm (complete data visualization toolbox, ggplot2/R-like)” toolbox from MATLAB File Exchange.

### Cell culture

U87 and A172 human GBM cells (ATCC) were cultured in DMEM (Life Technologies) supplemented with 10% fetal bovine serum (Sigma-Aldrich), 100 units/ml penicillin-100μg/ml streptomycin (Sigma-Aldrich). The cultures were maintained at 37 °C in a humidified atmosphere containing 5% CO_2_. For hypoxia studies, cells were cultured in 1% O_2_, 94% N_2_ and 5% CO_2_ (hypoxia) in a hypoxia chamber (BioSpherix, Xvivo system, G300C, NY).

### Glioblastoma multiforme (GBM) tissue array

Glioblastoma multiforme (GBM) tissue array (GL805a) was purchased from US BioMax (MD, USA). It consisted 35 cases of glioblastoma tissue and 6 cases of normal brain tissues as control. Immunohistochemistry (IHC) analyses were performed on a Bond Rx autostainer (Leica Biosystems, Germany) with heat mediated antigen retrieval (Bond ER1) using sequential double staining protocol. Antibodies used were mouse anti-human BIRC3 (R&D Systems MAB817, 1:600) and rabbit anti-human CA9 (Novus Biologicals NB100–417SS, 1:1500). The enzymes horseradish peroxidase (HRP) and alkaline phosphatase (AP) were used for detection. Sections were counterstained with hematoxylin, dehydrated and the slides coverslipped using a TissueTek-Prisma and Coverslipper (Sakura, USA). Whole slide scanning (40x) was performed on an Aperio AT2 (Leica Biosystems, Germany). The IHC staining was performed by Histowiz (NY, USA).

### Real-time PCR

Total RNA was extracted using TRIzol (Life Technologies). RNA was quantified with Nanodrop 2000 (Thermo Scientific). cDNA was synthesized using 1 μg total RNA with the iScript cDNA Synthesis Kit (Bio-Rad). Real-time PCR was performed using the Bio-Rad CFX96 Touch Real-Time PCR Detection system. *HIF-1α* forward primer: CCAGCAGACTCAAATACAAGAACC,reverse primer: TGTATGTGGGTAGGAGATGGAGAT; *BIRC3* forward primer: AGCTACCTCTCAGCCTACTTT; reverse primer: CCACTGTTTTCTGTACCCGGA. *β-actin* was used as the internal control; *β-actin* forward primer: TCCTGTGGCATCCACGAAACT, reverse primer: GAAGCATTTGCGGTGGACGAT. The PCR program was as follows: 95 °C 10 minutes, 1 cycle; 95 °C 15 seconds −>60 °C 30 seconds −>72 °C 30 seconds, 40 cycles; 72 °C 10 minutes, 1 cycle.

### Western blot analysis

40 μg of heat-denatured proteins were loaded on 4–15% precast polyacrylamide gel (Bio-Rad). The proteins were then transferred to PVDF membranes (Bio-Rad), which were blocked with 5% non-fat milk solutions for 1 hour at room temperature. The target proteins were then detected by the primary antibody at 4 °C overnight, washed with 0.1% Tween-PBS and incubated with appropriate secondary antibody for 2 hours. The membranes were then washed and the target proteins were detected with luminol reagent and X-ray film (Santa Cruz). Quantification of the target protein was done using Adobe Photoshop. In brief, the background of the target protein and β-actin were subtracted. Then, the relative expression of the target protein was normalized to β-actin and compared to that of the control group in each experiment. Anti-human HIF-1α antibody was from Novus-bio. Rabbit anti-human BIRC3, goat anti-Rabbit IgG-HRP and anti-β-actin IgG-HRP were obtained from Santa Cruz Biotech.

### siRNA transfection

U87 and A172 GBM cells were transfected with BIRC3 small interfering RNA (siRNA; NM_001165 and NM_182962, Sigma-Aldrich, USA), HIF-1α siRNA (Santa. Cruz, sc-35561, USA; Thermofisher, 4390824) or control siRNA using Lipofectamine RNAiMAX (Life Technologies, Carlsbad, CA, USA). Briefly, one day prior to transfection, the cells were cultured in T25 flasks (5 × 10^5^) or 96 well plate (1 × 10^4^) with 10% FBS DMEM without antibiotics. An siRNA-lipofectamine complex mixture in serum-free Opti-MEM (Life Sciences) was prepared according to the manufacturer’s instructions and was added to the cells. The medium was replaced with DMEM containing 10% FBS 5 hours after transfection.

### Chromatin immunoprecipitation (ChIP) assay

The CHIP assay was done using the Pierce Agarose CHIP Kit (Cat. 26156, Thermo Scientific). Detailed protocol can be found at: https://tools.thermofisher.com/content/sfs/manuals/MAN0016149_PierceAgaroseChipKit_PI.pdf. The eluted DNA was used as the template for *BIRC3* promoter PCR. Forward primer: 5′-GGGCATATTGACCTTTTCCA-3′; Reverse primer: 5′-AAATCCCCACCCCTATCTGT-3′. Thermocycler Amplification Settings: Step 1: 95 °C for 15 minutes. Step 2: 95 °C for 15 seconds. Step 3: 62 °C for 1 minutes. Step 4: Repeat Steps 2 to 3 for 40 cycles. PCR products were detected by 4% agarose gel electrophoresis.

### X-ray irradiation

Cells were irradiated using XRAD 160(Precision X-ray Inc) at the rate of 2.5 Gy/min.

### Survival assay

Cell survival was measured using an XTT Cell Viability Assay Kit (Cell Signaling). In brief, following treatment/transfection, 50 µL of XTT detection solution was added to each well of a 96-well plate (containing 200 µL/well of culture medium) and incubated at 37 °C for 1 hours. The absorbance was then measured at 450 nm using a microplate reader (Molecular Device). The relative survival was calculated by dividing the absorbance of experimental group with that of the control group.

### Caspase3/7 activation assay

Caspase3/7 activation was detected using CellEvent™ Caspase-3/7 Green Flow Cytometry Assay Kit (Life Science, USA). Samples were read on BD LSRII and analyzed with FlowJo (Tree Star,USA).

### U87 GBM xenograft model and immunohistochemistry

Two million U87 GBM cells were injected subcutaneously in the flank of female NCRNU athymic mice. After four weeks mice were sacrificed and tumor samples were removed and fixed with 10% neutral-formalin buffer for 24 hours. The samples were then dehydrated, paraffin-embedded and sectioned (4 μm). Sections were dewaxed, treated with 3% H_2_O_2_ for 10 min and incubated with anti-BIRC3 antibody (R&D system, 1:600), anti-CA9 antibody (Novusbio,1:1500) or anti-HIF-1α (Novusbio,1:100) overnight at 4 °C. Biotinylated secondary antibody (1:200 dilutions) was added at room temperature for 1 hr, followed by the incubation with ABC-peroxidase for additional 1 hour. After washing with Tris-buffer, the sections were incubated with DAB (3,30 diaminobenzidine, 30 mg dissolved in 100 ml Tris-buffer containing 0.03% H_2_O_2_) or other substrate for 5 minutes, rinsed in water and counterstained with hematoxylin. IHC staining was done by Histowiz (NY).

### Statistics

For the bioinformatics gene expression analysis, group comparisons were performed using Mann-Whitney test and the two-tailed p-value is reported. Fold change is reported as fold change (fc). Box plots and scatter plots were generated using MATLAB R2016a (TheMathWorks, Natick, MA, USA) and the “Alternative box plot” toolbox and “gramm (complete data visualization toolbox, ggplot2/R-like)” toolbox from MATLAB File Exchange. For biological replicates, the student’s t-test was used and p < 0.05 was considered significant difference.

### Data availability

The datasets generated and/or analysed during the current study are available in the [TCGA] repository. https://cancergenome.nih.gov/.

## Results

### BIRC3 gene expression is a selective biomarker for the mesenchymal GBM subtype

**Figure 2:**
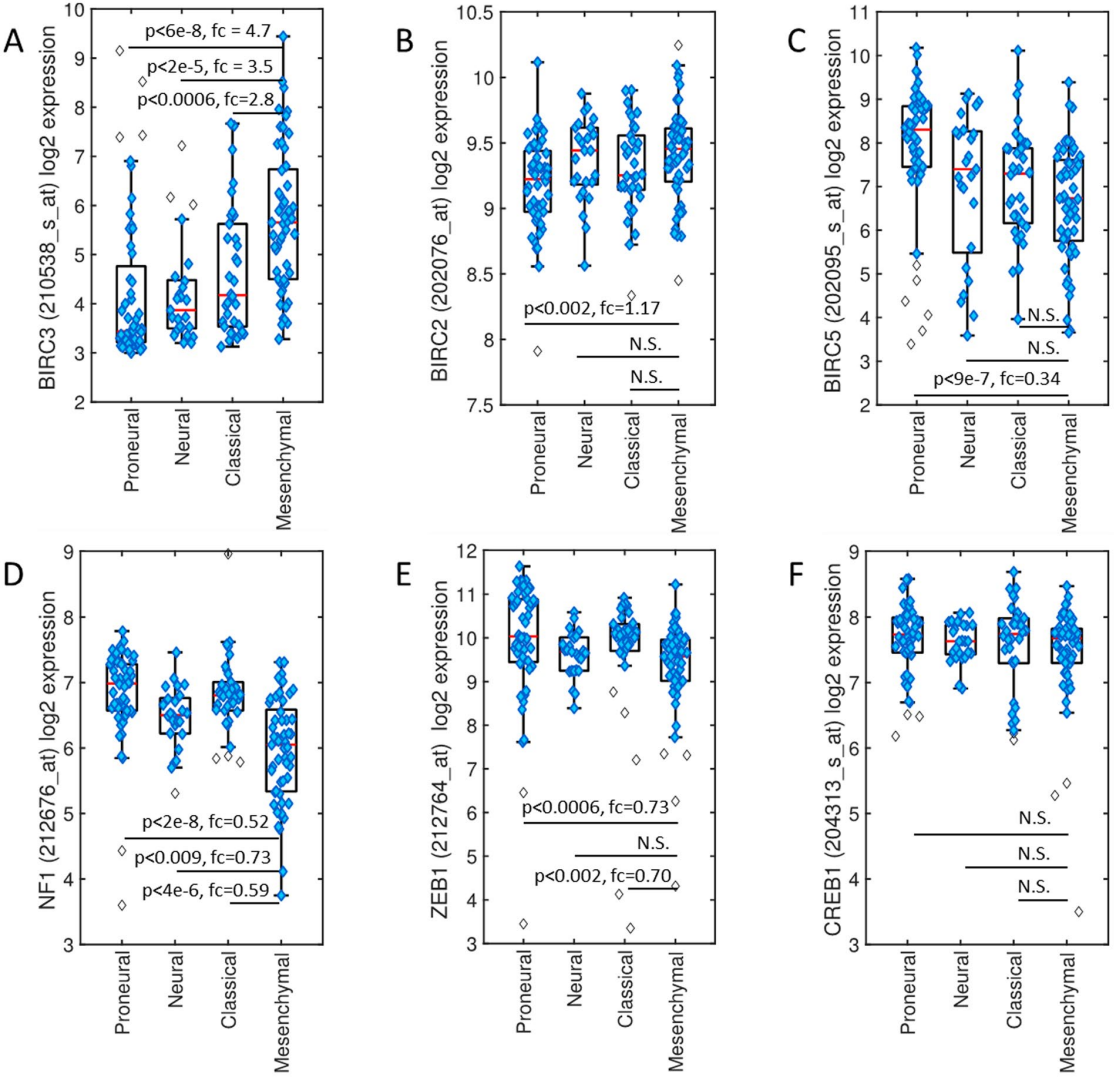
*BIRC3* expression segregates mesenchymal GBM from the other GBM subtypes. Normalized log2 expression was compared across the neural, proneural, classical, and mesenchymal GBM subtypes. Results are represented in box plot format: (**A**) *BIRC3*; (**B**). *BIRC2*; (**C**) *BIRC5*; (**D**) NF1; (**E**) *ZEB1*; and (**F**) *CREB1*.

Gene expression arrays, copy number data and mutational analyses of TCGA datasets have defined the four subtypes – neural, proneural, classical and mesenchymal – of GBM^10^. However, TCGA subtyping of GBM is not in practice in the clinic. The mesenchymal GBM subtype is the most aggressive and difficult to treat. As we had shown that BIRC3 can promote therapeutic resistance in GBM^31^, BIRC3 expression was assessed in GBM subtypes. Normalized expression analysis were performed using the U133A platform using 173 TCGA GBM samples previously classified on the basis of subtypes. With this data there were proneural (n = 52), neural (n = 25), classical (n = 34), and mesenchymal (n = 53) GBM samples. Across GBM subtypes, *BIRC3* levels were significantly elevated in mesenchymal GBM versus other subtypes (Fig. 2A). There was actually at least a 5-fold elevation of BIRC3 levels in mesenchymal compared to pro-neural GBM (p < 0.00000006). In contrast, there were no significant differences in the expression of BIRC2 or BIRC5 between GBM subclasses (Fig. 2B–C). Analysis of the expression of other mesenchymal gene candidates such as *NF1* ^10^, *CREB1* ^34^, and *ZEB1* ^26, 35–37^ across GBM subtypes revealed that *NF1* and *ZEB1* expression were rather significantly reduced in mesenchymal versus other GBM subtypes, whereas CREB1 expression was similar across GBM subclasses (Fig. 2D–F). Thus, elevated BIRC3 is a discrete segregator of mesenchymal GBM.

Analyses of other members the mesenchymal gene cluster employed in the TCGA subclass analysis^10^, including *CASP1*, *IL4R*, *CHI3L1*, *TRADD*, *TLR2* and *RELB* (Supplementary Figure 1), revealed that these also segregated towards the mesenchymal subtype similar to BIRC3. This was very interesting since the previous TCGA subclass analysis^10^ of GBM never identified BIRC3 as a mesenchymal habitat segregator. One possible explanation as to why mesenchymal regions in GBM might have a propensity for higher BIRC3 expression is that GBM mesenchymal transformation has been reported to be mediated via NF-κB^38^, which has known links with BIRC3^39, 40^.

### BIRC3 gene expression in mesenchymal GBM Habitats is independent of other known mesenchymal GBM genes

**Figure 3:**
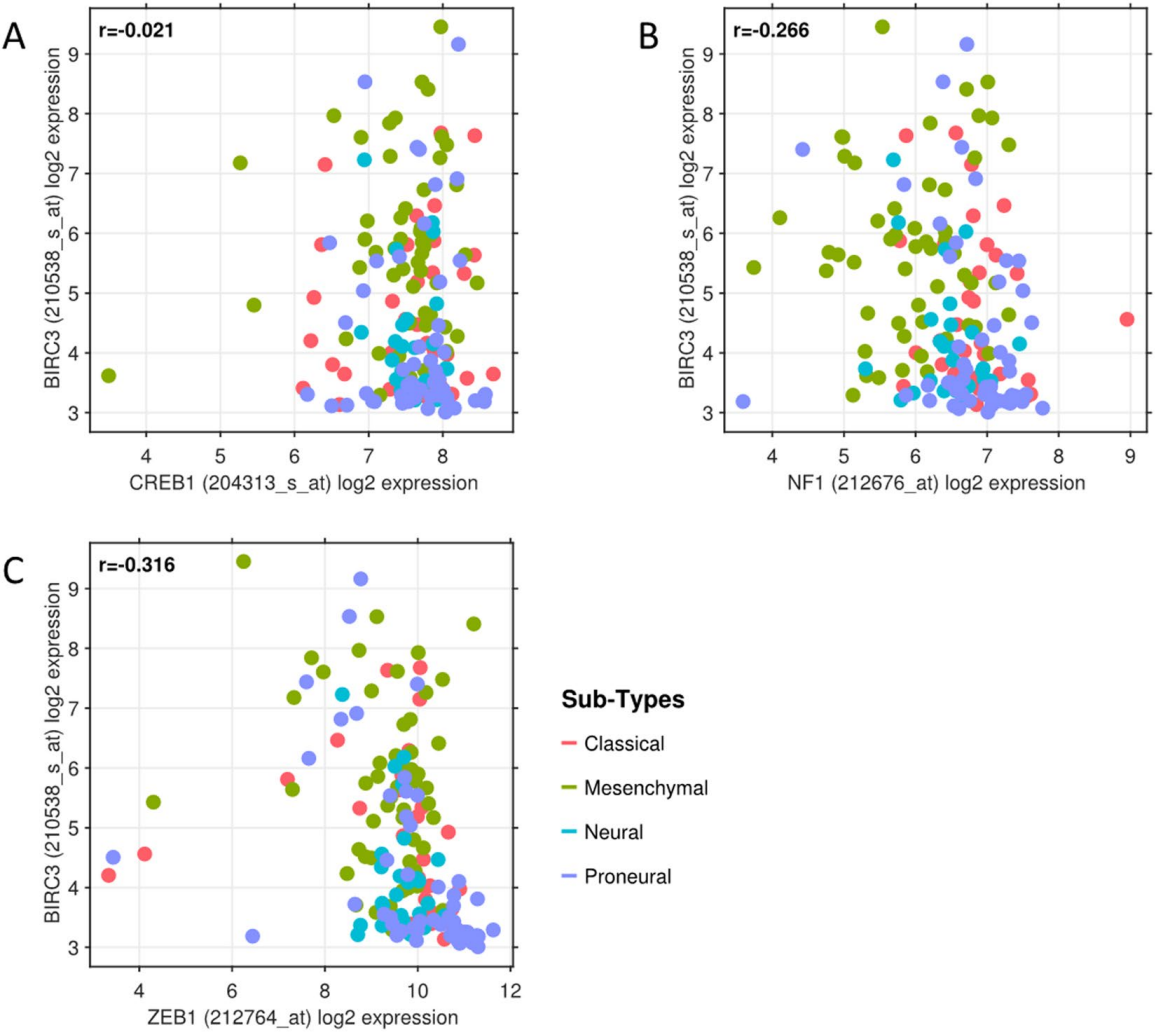
*BIRC3* expression is a unique identifier of mesenchymal GBM. Normalized log2 expression of *BIRC3* was plotted against other proposed mesenchmymal-selective genes in a scatter plot, colored by GBM subtypes and with *r* representing Pearsons correlation coefficient. (**A**) *BIRC3* versus *CREB1* expression. (**B**) *BIRC3* versus *NF1* expression. (**C**) *BIRC3* versus *ZEB1* expression.

As elevated levels of BIRC3 were a selective hallmark of mesenchymal GBM, we sought to determine if BIRC3 expression is truly distinct or correlated with expression of other mesenchymal-subtype signature genes. To eliminate bias we examined the correlation between BIRC3 expression and several genes across all GBM subtypes. The comparison of *BIRC3* expression to changes in *NF1*, *CREB1*, and *ZEB1* expression across all GBM subtypes failed to reveal any correlation between *BIRC3* expression and *NF1*, *CREB1*, or *ZEB1* (Fig. 3A–C). Further, a similar analysis examining correlation between *BIRC3* expression and TCGA mesenchymal cluster genes *CASP1*, *IL4R*, *CHI3L1*, *TRADD*, *TLR2* and *RELB* ^10^ failed to demonstrate any correlation (Supplementary Figure 2A–F). These findings suggest that, at least from a statistical standpoint, *BIRC3* expression in mesenchymal habitats is distinct, and perhaps independent from, the expression of previously described TCGA mesenchymal cluster genes^10^.

### BIRC3 protein is highly expressed uniformly in the tumor cell niche compared to the vascular endothelial niche across GBM regions

**Figure 4:**
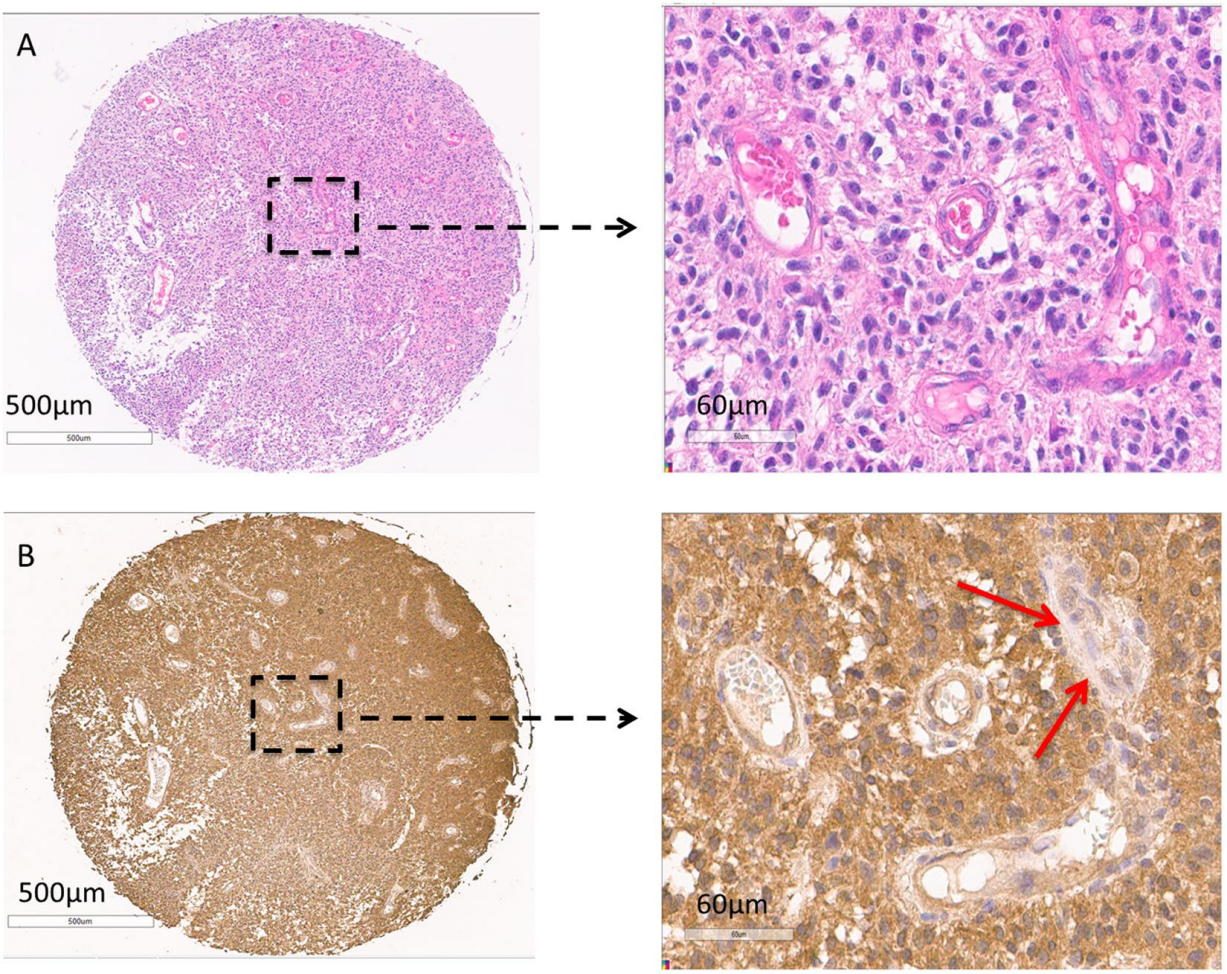
*BIRC3* is expressed at higher level in the tumor cell niche compared to the vascular niche in GBM. Human GBM tissue microarray was stained for BIRC3 (Brown). BIRC3 expression can be compared between GBM tumor cell niche and vascular endothelial cell niche (**A,B**). Please note the focus of microvascular proliferation which shows negligible BIRC3 expression (Red arrow).

Although BIRC3 gene expression data from the TCGA revealed differential yet positive expression of BIRC3 across all subtypes of GBM, the relative contributions of BIRC3 protein expression by the tumor cell niche and vascular microenvironment niche remains unclear. Furthermore, hypoxia is highly featured in GBM and remains a major factor in GBM survival adaptations^14^ and promotes mesenchymal transformation^26^. We were therefore interested in characterizing regional expression of BIRC3 protein in GBM using patient GBM tissue microarray with respect to tumor cell niche as well as vascular niche. We examined regions of vascular proliferation as well as pseudopalisading necrosis both of which are recognized sequela of hypoxia in GBM^15, 16^. To assess BIRC3 protein expression in tumor cell niche versus vascular niche, we performed immunohistochemistry (IHC) analyses of a TMA comprised of patient-derived GBM biopsy samples as well as normal brain controls. Notably, BIRC3 was uniformly expressed across all regions in GBM specimens including areas with endothelial proliferation and pseudopalisading necrosis (Fig. 4A,B). Further, there was a relatively higher expression of BIRC3 in the tumor cell niche compared to the vascular niche (Fig. 4A,B) suggesting that the tumor cells are largely responsible for BIRC3 expression. There was negative/equivocal BIRC3 expression in areas of glomeruloid proliferation, in stark contrast to the surrounding tumor (arrowheads Fig. 4A,B) demonstrate negative/equivocal The stark contrast between BIRC3 expression. It should be noted that the GBM samples in the TMAs were never characterized for TCGA subtype stratification. Nonetheless, BIRC3 protein expression appeared to be a robust habitat marker in GBM with a predilection towards the tumor cell niche compared to the vascular niche.

### Hypoxia induces BIRC3 expression in glioblastoma

**Figure 5:**
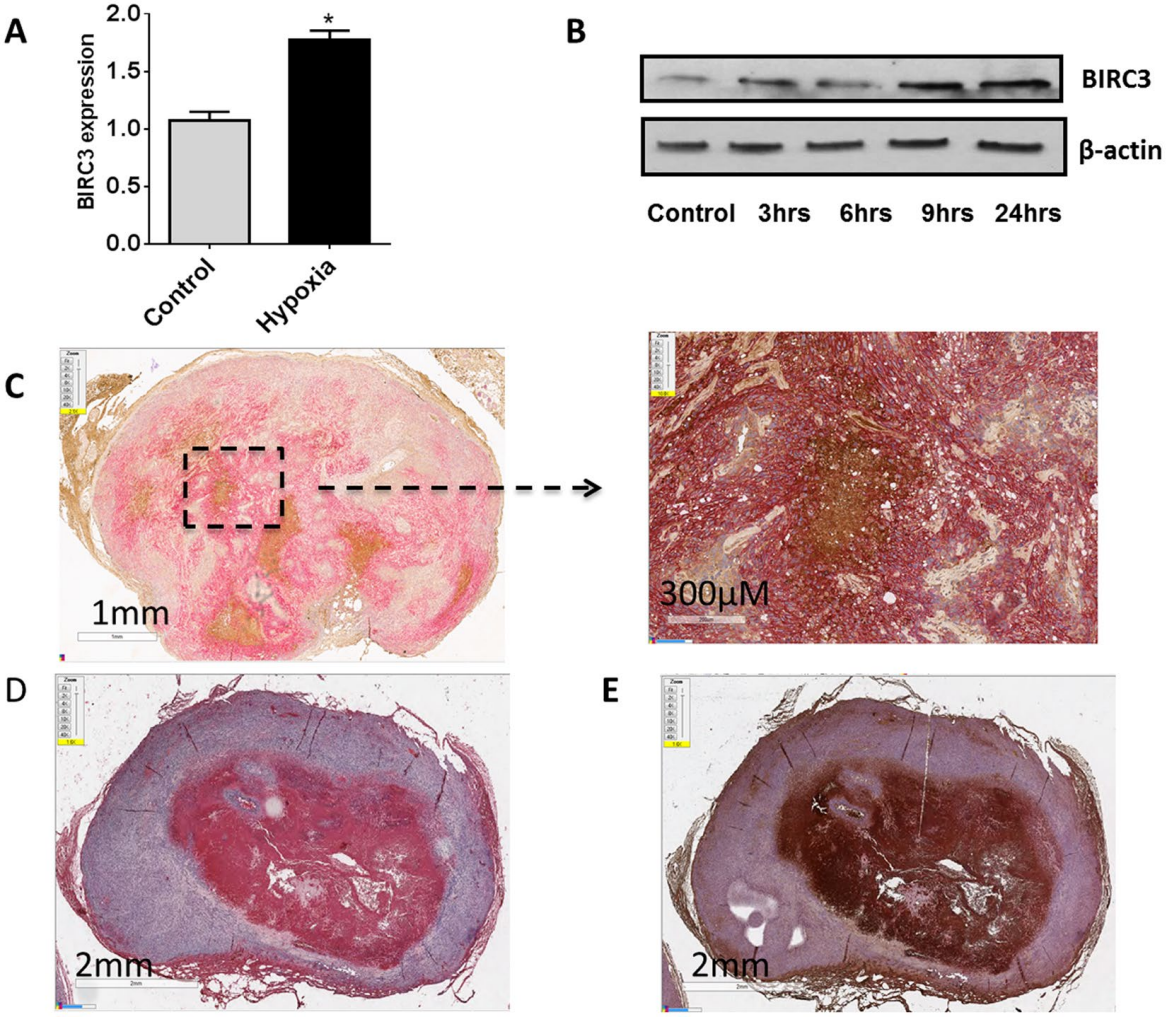
Hypoxia induces *BIRC3* expression in GBM *ex vivo* and *in vivo*. (**A**) U87 GBM cells were cultured under hypoxic conditions (1% O_2_) for 24 hr and BIRC3 gene expression was analyzed by RT-PCR. Data are representative of three independent experiments (p < 0.05). (**B**) U87 cells were cultured under hypoxia conditions (1% O_2_) for the indicated intervals and BIRC3 protein levels were determined by western blot. Data are representative of three independent experiments. Whole images for Western-blot can be found in the Supplementary Figure 7. (**C**–**E**) GBM xenografts were established by by injecting 2 × 10^6^ U87 cells on the flank of 6–8 week *nude* mice. At 6 weeks, mice were sacrificed and xenografts were assessed for BIRC3 (brown) and CA9 (pink) or HIF-1α (pink) expression by IHC. (**C**) BIRC3 and CA9 expression. (**D**) HIF-1α expression. (**E**) BIRC3 and HIF-1α expression.

To determine if there was a causal relationship between hypoxia and BIRC3 expression, we first assessed *BIRC3* expression levels by qRT-PCR in U87 GBM cell lines exposed to hypoxia. There were significant increases BIRC3 mRNA and protein levels when GBM cells were subjected to hypoxia (Fig. 5A,B **;** p < 0.05). We used a second GBM cell line, A172 GBM to confirm our hypoxia findings noted with U87 GBM cells. Similarly, when A172 GBM cells were subjected to hypoxia, we observed BIRC3 upregulation at the mRNA and protein levels (Supplementary Figure 3). To evaluate if increases in BIRC3 were also manifest in hypoxic regions of GBM *in vivo*, U87 human GBM xenografts were established in the flank of *nude* mice and tumors were analyzed by IHC. Notaby, there were high levels of BIRC3 (brown) in hypoxic regions of these xenografts, which were detected with Carbonic Anhydrase-9, CA9 (pink) antibody (Fig. 5C). Conversely, BIRC3 expression was less pronounced in non-hypoxic regions of these tumors. This phenotype was manifest across several regions in five different xenografts (Supplementary Figure 4). There was also concordant expression of HIF-1α and BIRC3 in these xenografts (Fig. 5D,E). Overall, these findings suggest that regional variations in hypoxia drive BIRC3 expression in GBM and that BIRC3 expression defines hypoxic regions in GBM that are islands of resistance in these tumors.

### Inhibition of HIF-1α blocks hypoxia-directed up-regulation of BIRC3 in GBM

**Figure 6:**
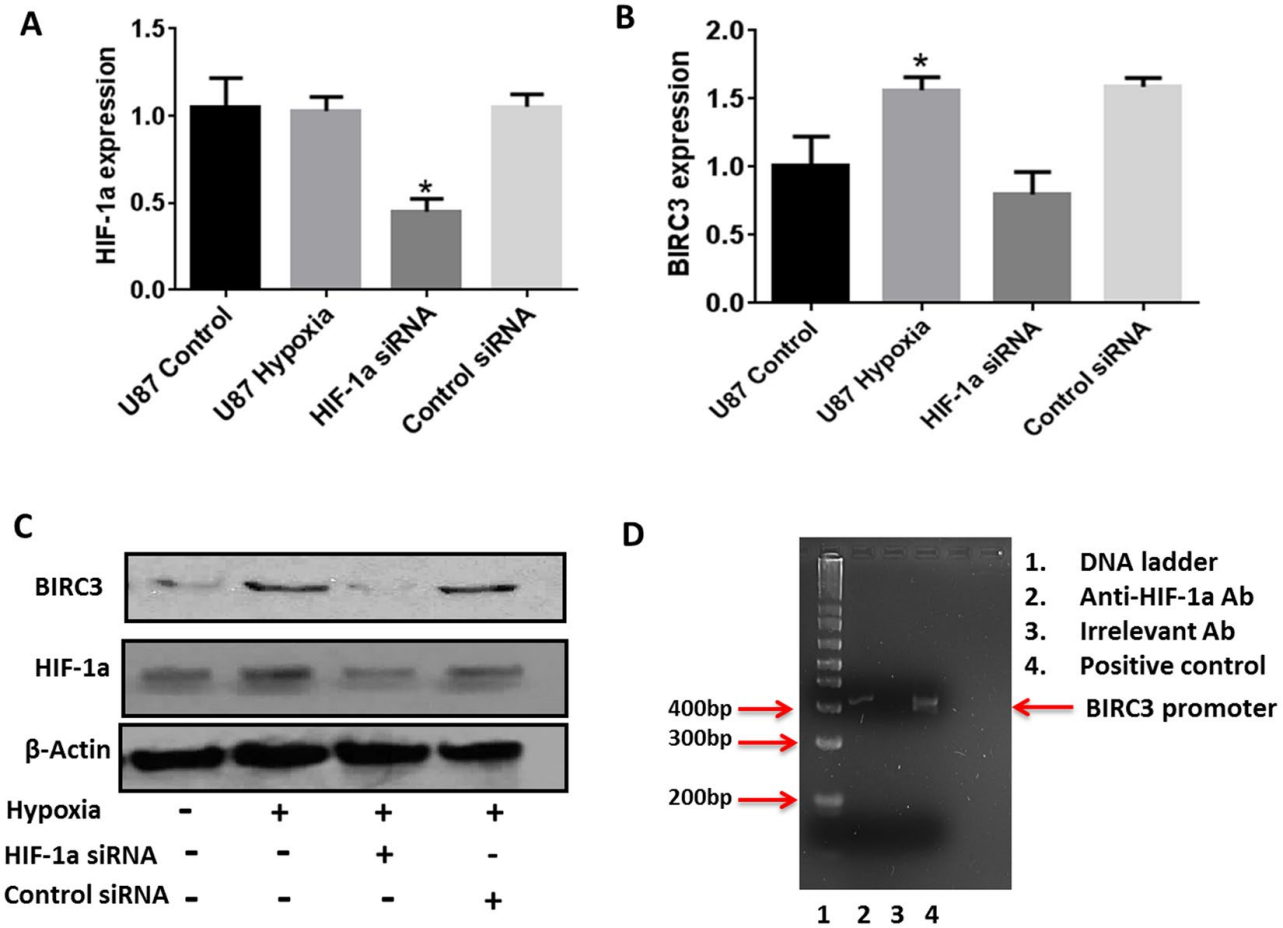
Inhibition of HIF-1α blocks hypoxia-induced up-regulation of *BIRC3* expression in GBM. (**A,B**) U87 GBM cells, or these cells transfected with HIF-1α siRNA for 48 hr, were cultured under hypoxia conditions (1% O_2_) for 24 hr. Efficiency of HIF-1α knockdown and effects on BIRC3 gene expression were determined by qRT-PCR (n = 3 independent experiments, p < 0.05). **(C)** BIRC3 protein level were also assessed following knockdown of HIF-1α + /− hypoxia (n = 3 independent experiments). Whole images for Western-blot can be found in the Supplementary Figure 8. (**D**) ChIP of HIF-1α on the *BIRC3* gene promoter was performed in U87 GBM cells exposed to hypoxia (1% O_2_ for 24 hr).

We next tested if HIF-1α was necessary for hypoxia-mediated increases in BIRC3 expression in GBM. As expected^41, 42^, exposure of GBM cells to hypoxia led to increases in HIF-1α protein but not mRNA (Fig. 6A,C; p < 0.01). Notably, selective knockdown of HIF-1α with siRNA (Fig. 6A,C) significantly impaired hypoxia-mediated induction of BIRC3 mRNA and protein levels in U87 GBM cells (Fig. 6B,C; p < 0.01). Finally, chromatin immunoprecipitation (ChIP) analyses established that HIF-1α selectively bound to the BIRC3 promoter in hypoxia treated U87 GBM cells (Fig. 6D). We used a second GBM cell line, A172 GBM to confirm our HIF-1α hypoxia signaling findings noted with U87 GBM cells. Similarly, HIF-1α was implicated in hypoxia mediated upregulation of BIRC3 in A172 GBM cells through siRNA silencing with two distinct HIF-1α targeting siRNAs and promoter interaction analysis (Supplementary Figure 5).

### Silencing BIRC3 impairs hypoxia-induced resistance of GBM to radiotherapy through enhancement of caspase activation and activity

**Figure 7:**
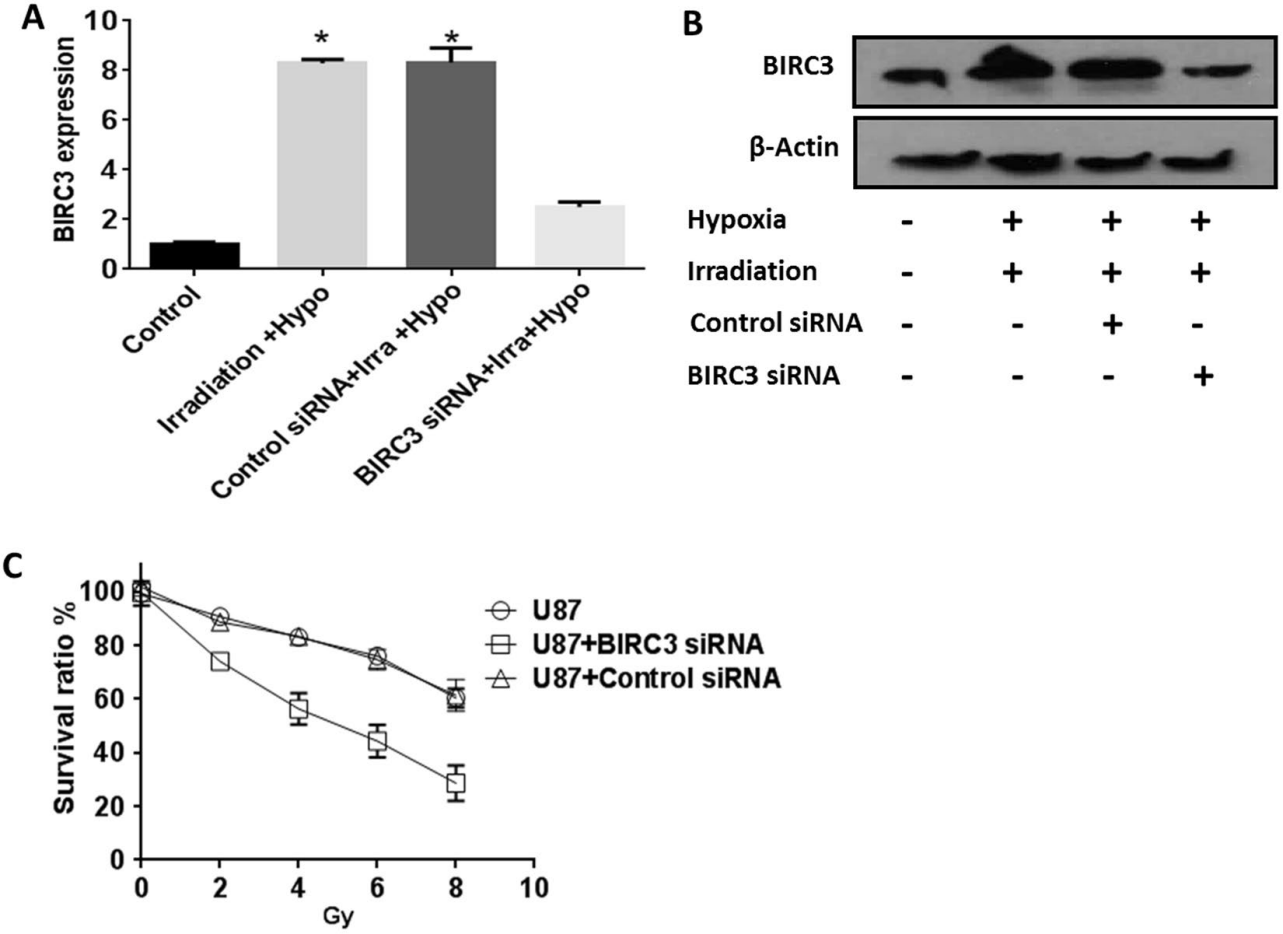
*BIRC3* silencing impairs hypoxia-induced survival of GBM to radiotherapy (RT). 1 × 10^4^ U87 MG GBM cells were cultured in 96 well plate under hypoxia (1% O_2_) condition for 12 hr and irradiated with 4 Gy. Cells were returned to hypoxia conditions for another 12 hr and harvested. BIRC3 mRNA and protein expression were analyzed by RT-PCR (**A**) and Western blot, respectively (**B**). Similar results were obtained from three independent experiments (p < 0.05). Whole images for Western-blot can be found in the Supplementary Figure 9. (**C**) U87 GBM cells with or without BIRC3 siRNA pretreatment (48 hr earlier) were cultured under hypoxia (1% O_2_) for 12 hr and irradiated with 2 Gy, 4 Gy, 6 Gy or 8 Gy. Cells were returned to hypoxia conditions for another 24 hr and cell survival were assessed using an XTT Cell Viability Assay Kit. Data are representative of three independent experiments (p < 0.05).

It is well established that hypoxia can render GBM cells resistant to radiotherapy (RT)^43, 44^. To test if BIRC3 was necessary for hypoxia-induced resistance to RT, U87 GBM cells were treated with BIRC3-specific siRNA, which significantly impaired BIRC3 mRNA and protein levels in cells exposed to hypoxia conditions versus control siRNA treated cells (Fig. 7A,B **;** p < 0.05). Notably, silencing BIRC3 augmented the sensitivity of GBM cells treated with RT under hypoxic conditions (Fig. 7C). We used a second GBM cell line, A172 GBM to confirm the role of BIRC3 in hypoxia-induced resistance of GBM to RT similar to what we had observed with U87 GBM cells. Similarly, BIRC3 was implicated in hypoxia-induced resistance of GBM to RT in A172 GBM cells through siRNA silencing with two distinct BIRC3 targeting siRNAs (Supplementary Figure 6A–C). Since we had previously implicated BIRC3 as a driver of apoptosis evasion in GBM^31^, we sought to determine if a similar mechanism was responsible for its role in hypoxia-survival adaptation. We therefore examined the Caspase3/7 activation status in A172 GBM cells irradiated in either the presence or absence of BIRC3 targeting siRNA. Caspase activation was markedly enhanced through selective inhibition of BIRC3 expression in hypoxia (Supplementary Figure 6D). Taken together, these results suggest that BIRC3 plays a role in hypoxia-mediated survival adaptation through suppression of caspase activitation. In addition, reversal of BIRC3 expression increases sensitivity of GBM cells to RT in hypoxic conditions. Thus, BIRC3 is an attractive target for hypoxia-resistance in GBM.

## Discussion

Hypoxia-mediated survival adaptation is known to be a major mechanism of resistance to conventional or targeted therapeutics and to RT^2–9^ that drives the evolution of aggressive tumor phenotypes^45, 46^. Hypoxia-driven vascular proliferation and pseudopalisading necrosis are classic histopathologic features of GBM that are associated with an aggressive tumor phenotype, therapy resistance and lethality. Further, hypoxia is known to drive mesenchymal transformation in GBM^26^. However, heretofore the mechanisms by which hypoxia contributes to survival adaptation in hypoxic habitats in GBM have been unclear. Further, there is a dearth of specific tissue biomarkers of hypoxia-adaptive GBM cellular habitats that could inform the targeted delivery of therapeutics and an assessment of treatment response.

The present study highlights a key role of BIRC3 in hypoxia-mediated survival adaptive phenotype of GBM habitats. Importantly, differential gene expression of BIRC3 is revealed as a clinical biomarker of mesenchymal GBM habitat where BIRC3 is selectively highly expressed in mesenchymal GBM. Further, BIRC3 is revealed as a direct HIF-1α transcription target whose expression is induced in GBM in response to hypoxia both *ex vivo* and *in vivo*. Finally, BIRC3 is shown to be a key mediator of hypoxia-mediated survival adaptation of GBM to RT through inhibition of caspase activation, suggesting that agents that disable this apoptotic regulator could have activity against mesenchymal GBM, which has dismal prognosis.

The identification of elevated BIRC3 mRNA and protein as a defining feature of mesenchymal GBM marker is novel and, interestingly, our analyses indicate that it is an independent biomarker of this subtype, as it does not correlate with alterations in the expression of NF-1, and other genes such as CASP1/4/5/8, ILR4, CHI3L1, TRADD, TLR2/4, and RELB^10^ that have been associated with mesenchymal GBM. In part, the unique association of BIRC3 expression as a biomarker of mesenchymal GBM might reflect selective up-regulation in hypoxic regions of mesenchymal GBM that also define this subtype. Regardless, a major advantage of BIRC3 as a potential biomarker of mesenchymal GBM is that subsequent IHC analyses with validated antibody to BIRC3 are likely sufficient to define this subtype in GBM pathology specimens.

Importantly, the findings presented herein establish that BIRC3 is a mediator of hypoxia-mediated survival adaptation in GBM, where: (i) hypoxia induces BIRC3 in a HIF-1α-dependent fashion; (ii) hypoxic regions of GBM xenografts have overlapping expression of HIF-1α and BIRC3; (iii) BIRC3 is a direct transcription target of HIF-1α; and (iv) BIRC3 contributes to hypoxia-mediated resistance to RT though mechanisms involving inhibition of caspase activation. While our findings clearly link BIRC3 to HIF-1α and hypoxia, they do not imply that BIRC3 is not also regulated by other means. For example, we have previously shown that PI3K and STAT3 could also contribute to the control of BIRC3 expression in GBM^31^. Functionally, BIRC3 contributes to GBM survival likely via its role in inhibiting the activation of caspases^47, 48^, yet it also promotes inflammatory processes via regulation of tumor-necrosis factor-alpha (TNF-α)/NF-κB signaling^39, 40, 49–51^. Hence, based on our results, BIRC3 may contribute to therapeutic resistance^31^ via blocking apoptosis and by provoking pro-inflammatory aggressive phenotypes within hypoxia-adaptive GBM habitats.

Finally, our findings suggest BIRC3 fulfills an unmeet need as a clinical tissue biomarker for hypoxia-adaptive regions of mesenchymal GBM. Intratumoral regional heterogeneity is a hallmark of GBM and this has profound implications for regional response to therapy^52^. Accordingly, identifying and validating tissue biomarkers that will permit modeling of regional evolutionary dynamics in GBM and that will identify regional resistance for clinical translation is a dire need, and this is particularly true for hypoxia-evolved GBM habitats that are highly resistant to therapy, including TMZ and RT. Our data support the notion that further studies validating the role of IHC for BIRC will meet these needs to allow for regionalized and personalized therapy of mesenchymal GBM.

## Electronic supplementary material

Supplementary infomation
