# Supplementary material for: BIRC3 is a biomarker of mesenchymal habitat of glioblastoma, and a mediator of survival adaptation in hypoxia-driven glioblastoma habitats
Source: Sci Rep. 2017 Aug 24;7:9350. doi: 10.1038/s41598-017-09503-8 (PMC5570925; doi:10.1038/s41598-017-09503-8)
Supplement: Supplementary file 1 — Supplementary infomation [file 41598_2017_9503_MOESM1_ESM.pdf]

BIRC3 is a biomarker of mesenchymal habitat of glioblastoma, and a mediator of survival adaptation in hypoxia-driven glioblastoma habitats

Dapeng Wang[1], Anders E. Berglund[2], Rajappa S. Kenchappa[1], Robert J. MacAulay[3], James J. Mulé[4], and Arnold B. Etame[1*]

Departments of [1]Neuro-Oncology, [2]Biostatistics and Bioinformatics,[3]Anatomic Pathology, and [4]Immunology, H. Lee Moffitt Cancer Center and Research Institute, 12902 Magnolia Drive, Tampa, FL 33612 USA

*Corresponding author:

Arnold B. Etame MD PhD
Department of Neuro-Oncology
Moffitt Cancer Center and Research Institute
12902 Magnolia Drive, Tampa, FL 33612
Tel: +1-813-745-3871
E-mail: arnold.etame@moffitt.org

Supplementary Figures

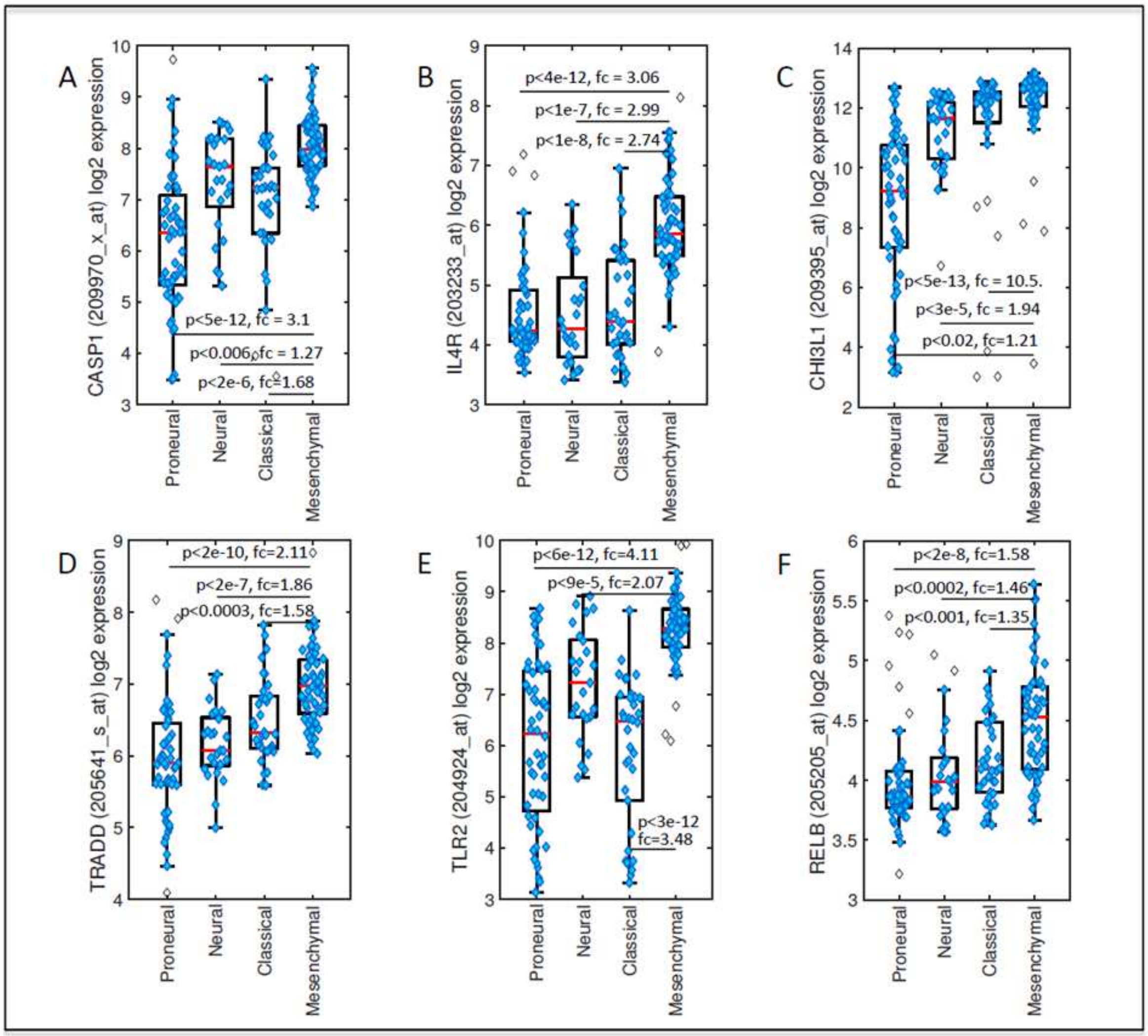

Supplementary Figure 1. TCGA mesenchymal gene expression cluster in GBM. The normalized log2 expression was compared across neural, proneural, classical, and mesenchymal GBM subtypes. Results are represented in box plot format: (**A**) *CASP1*; (**B**) *IL4R*; (**C**) *CHI3L*; (**D**)*TRADD*; (**E**) *TLR2*; and (**F**) *RELB*.

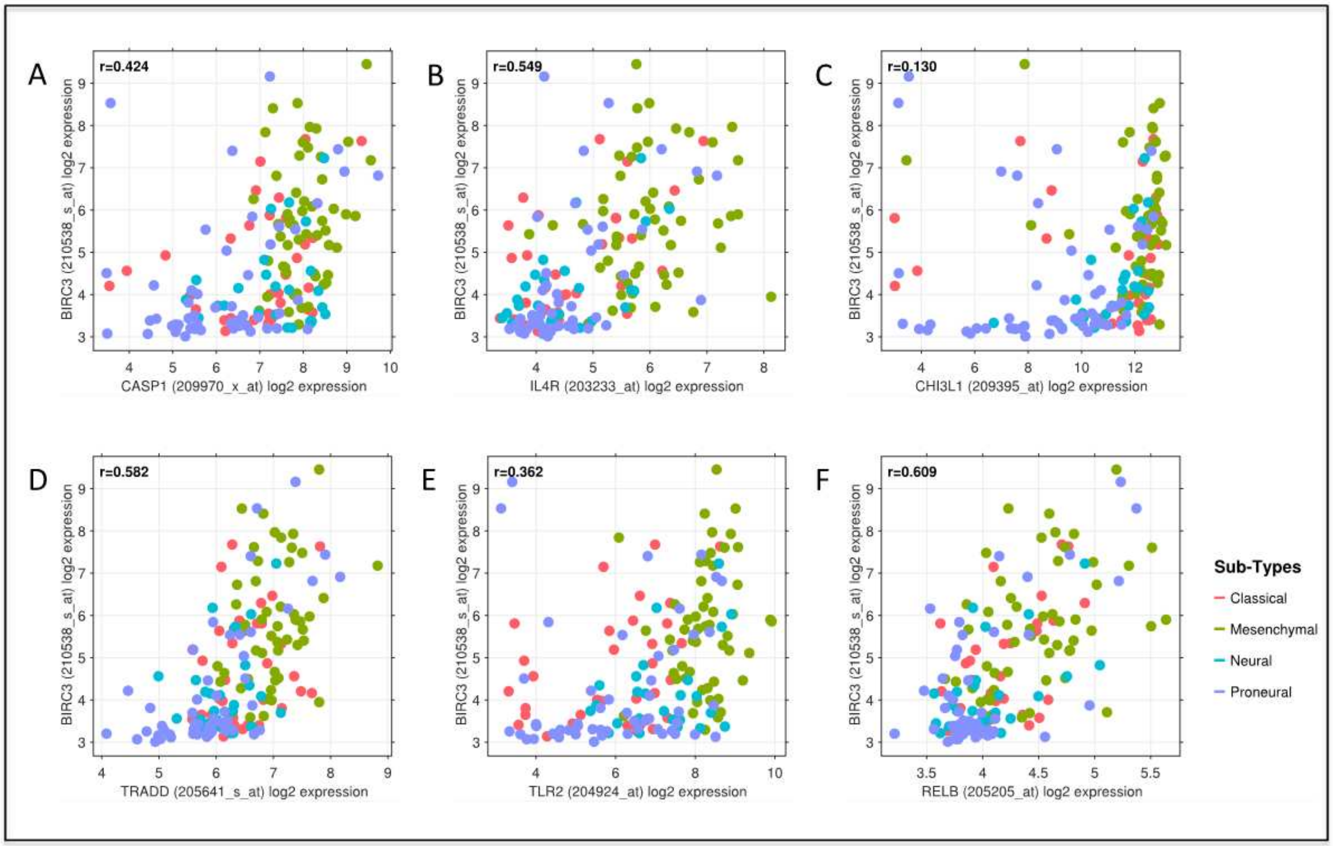

Supplementary Figure 2. BIRC3 gene expression is independent of the TCGA "mesenchymal gene expression cluster" in GBM. Normalized log2 expression of *BIRC3* was plotted against other genes in a scatter plot, colored by GBM subtypes and with *r* representing Pearsons correlation coefficient. (**A**) *BIRC3* versus *CASP1* expression. (**B**) *BIRC3* versus *IL4R* expression. (**c**) *BIRC3* versus *CHI3L1* expression. (**D**) *BIRC3* versus *TRADD* expression. (**E**) *BIRC3* versus *TLR2* expression. (**F**) *BIRC3* versus *RELB* expression.

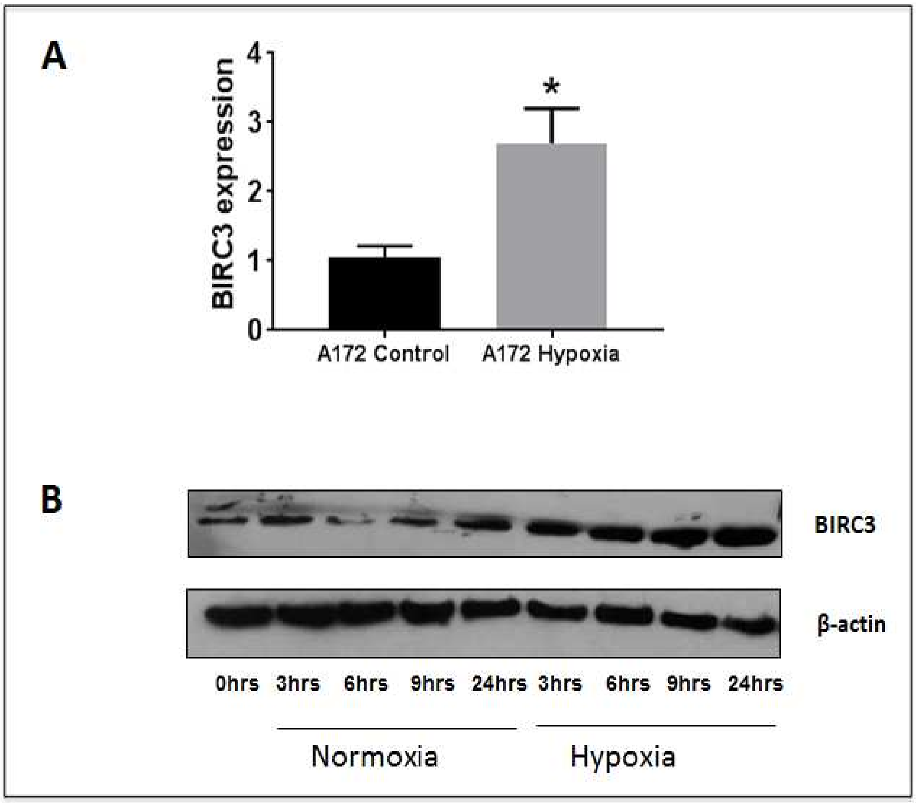

Supplementary Figure 3. Hypoxia drives BIRC3 expression in GBM. (**A**) A172 cells were cultured under hypoxia conditions (1% oxygen) for 24 hours and BIRC gene expression was analyzed by RT-PCR. Data are representative of three independent experiments. **p< 0.05.** (**B**). A172 cells were cultured under hypoxia conditions (1% oxygen). BIRC3 protein expression was compared at 3hour, 6hour, 9hour and 24hour by Western blot. Data are representative of three independent experiments.

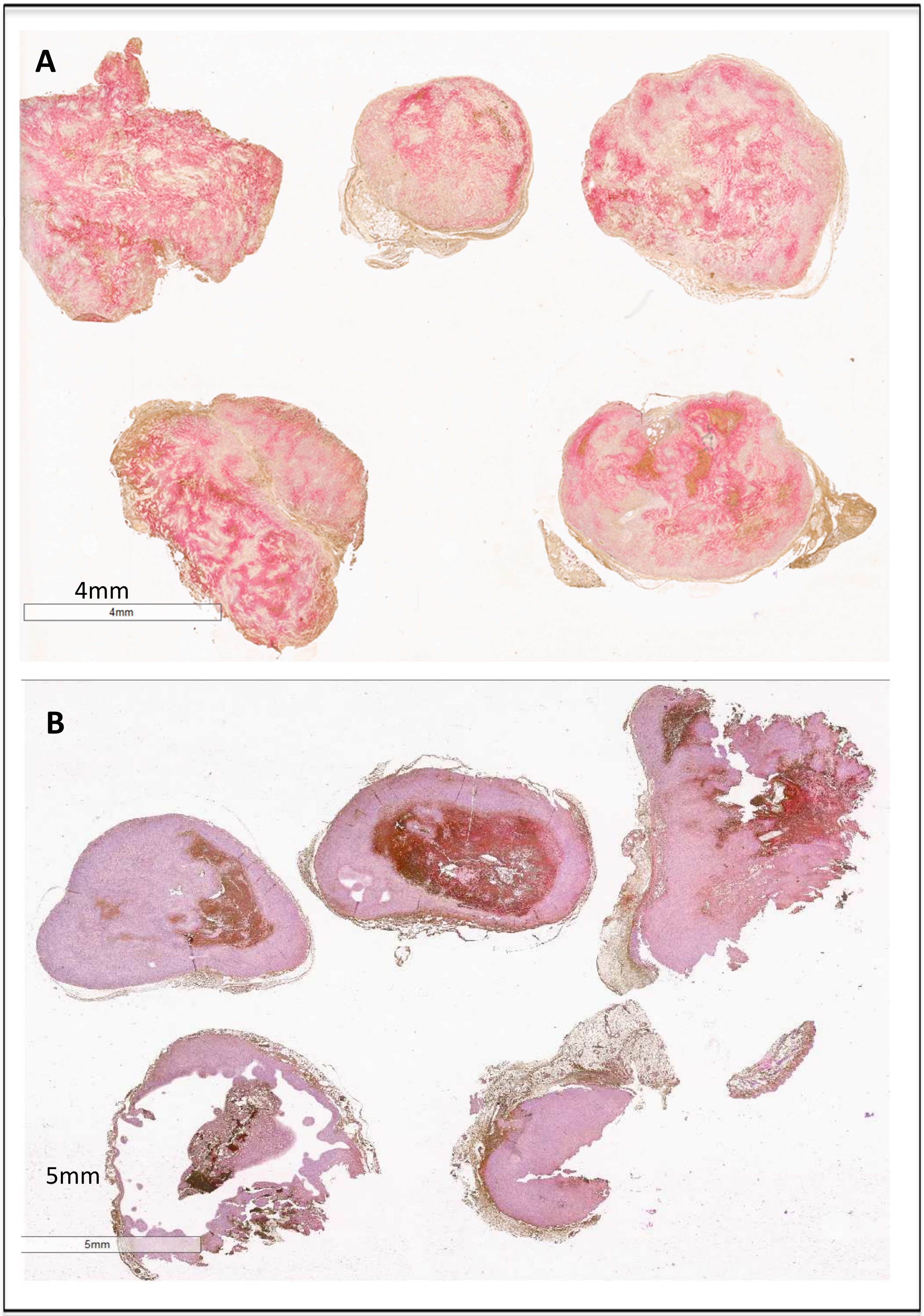

Supplementary Figure 4. **Hypoxic regions in GBM xenografts express elevated levels of BIRC3.** Mouse GBM xenografts were set up by injecting 2 × 10^6 U87 cells on the flank of 6-8 week nude mice. In 6 weeks, mice were sacrificed and xenografts were isolated for (**A**)BIRC3 (brown) and CA9 (pink) or (**B**)BIRC3 (brown) and HIF-1a (pink) immunohistochemistry study. Represented are 5 xenograft sections.

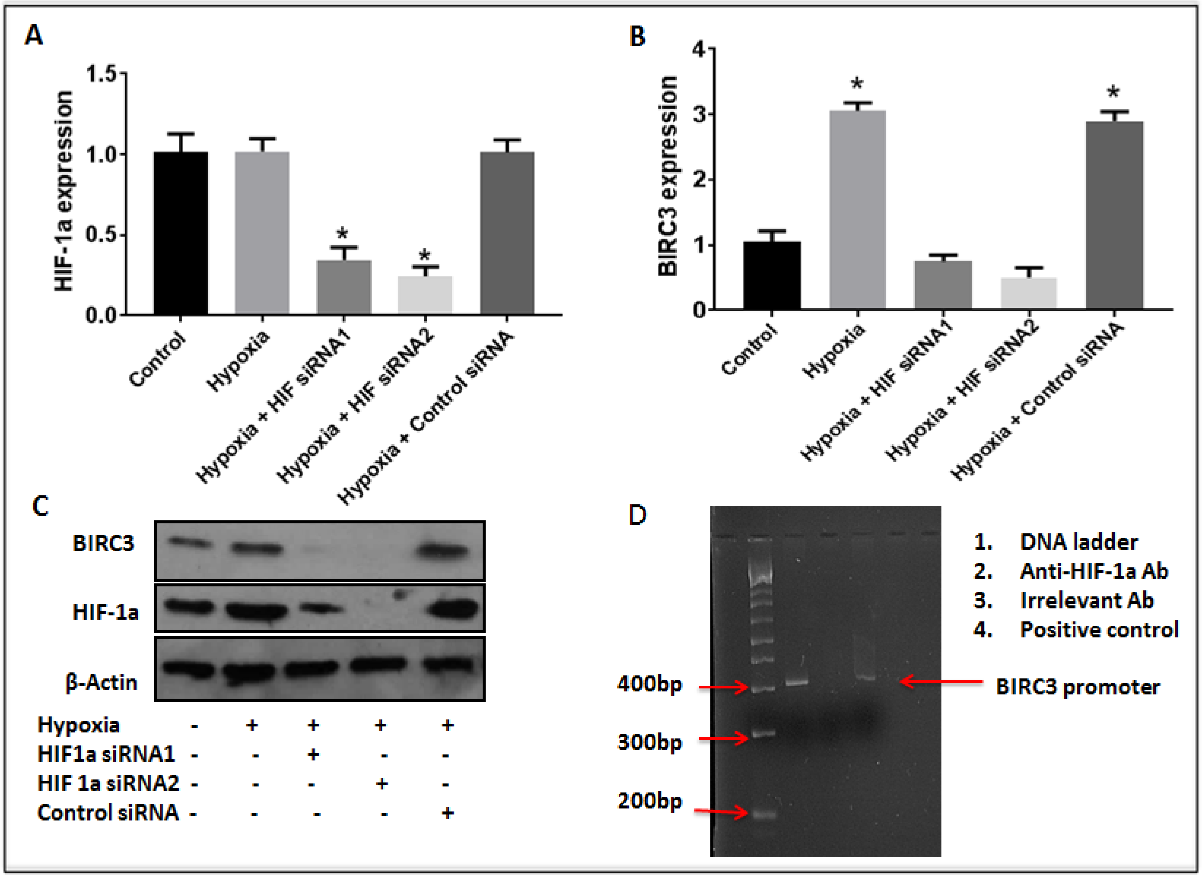

Supplementary Figure 5. Inhibition of HIF-1 Signaling Blocks Hypoxia-Induced Up-regulation of BIRC3 Expression in GBM. (A). A172 cells were cultured under hypoxia conditions (1% oxygen) for 24 hours with or without HIF-1a siRNA pretreatment (48hours earlier). HIF-1a gene expression was compared by RT-PCR**. (n=3 independent experiments, p<0.05).** (**B**). A172 cells were cultured under hypoxia conditions (1% oxygen) for 24 hours with or without HIF-1a siRNA pretreatment(48 hours earlier). **(C)** BIRC3 protein level were also assessed following knockdown of HIF-1α +/- hypoxia (n=3 independent experiments). (**D**). ChIP of HIF-1α on the *BIRC3* gene promoter was performed in A172 GBM cells exposed to hypoxia (1% O_2 for 24 hours). Lane 1: DNA ladder; Lane 2: BIRC3 promoter PCR using anti-HIF-1a antibody. Lane 3: BIRC3 promoter PCR using control antibody; Lane 4: BIRC3 promoter PCR positive control using human genomic DNA as template.

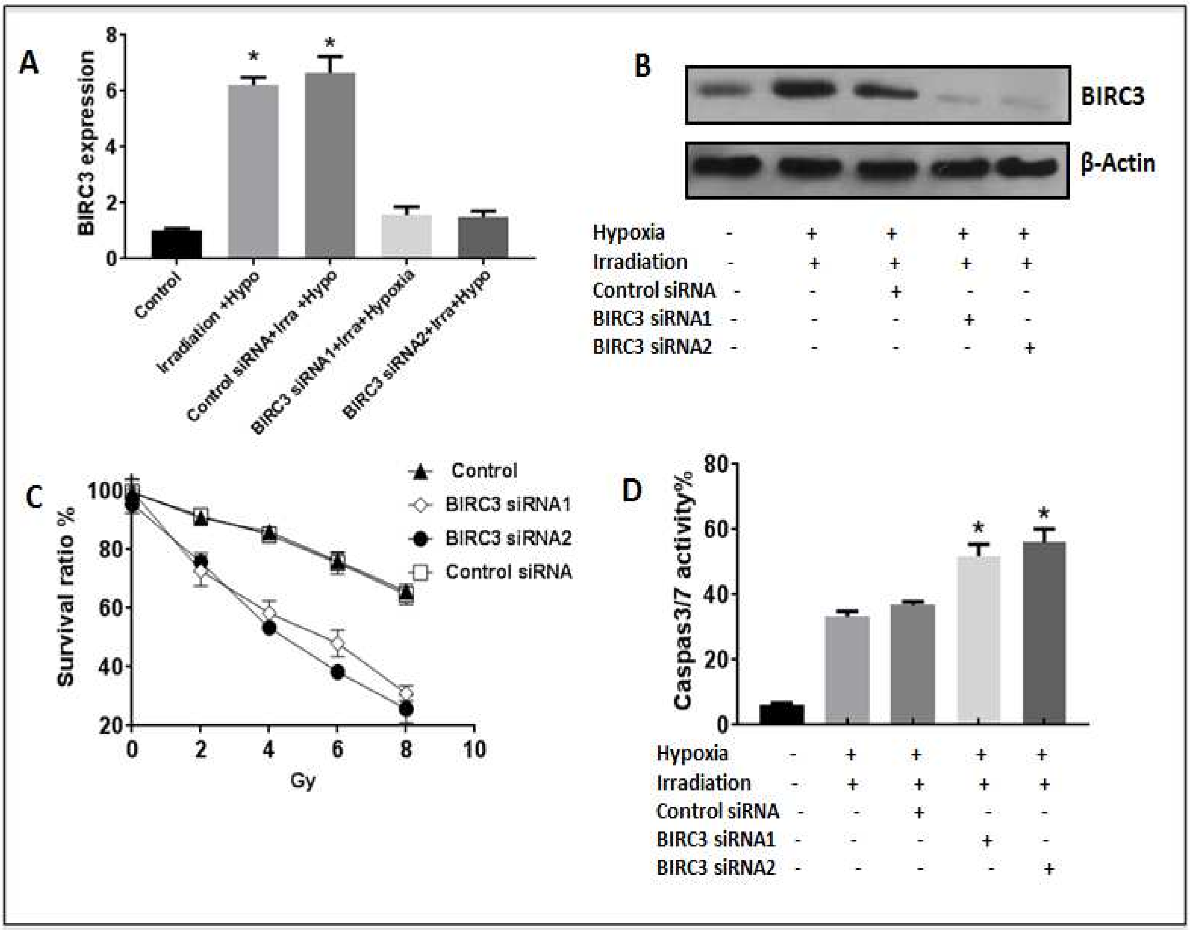

Supplementary Figure 6.Selective Inhibition of BIRC3 Reverses Hypoxia-induced Survival Adaptation of GBM to Radiotherapy. 1×10^4 A172 GBM cells were cultured in 96 well plate under hypoxia (1% O_2) condition for 12 hours and irradiated with 4Gy. Cells were returned to hypoxia conditions for another 12 hours and harvested. BIRC3 mRNA and protein expression were analyzed by RT-PCR (**A**) and Western blot, respectively (**B**). Similar results were obtained from three independent experiments ($p< 0.05$). **(C)** A172 GBM cells with or without BIRC3 siRNA pretreatment (48 hr earlier) were cultured under hypoxia (1% O_2) for 12 hour and irradiated with 2Gy, 4Gy, 6Gy or 8Gy. Cells were returned to hypoxia conditions for another 24 hr and cell survival were assessed using an XTT Cell Viability Assay Kit . Data are representative of three independent experiments ($p< 0.05$). (**D**). A172 GBM cells with or without BIRC3 siRNA pretreatment (48 hours earlier) were cultured under hypoxia (1% O_2) for 12 hours and irradiated at 4Gy. Cells were returned to hypoxia conditions for another 24 hours and Caspase3/7 activation were compared by flowcytometry.

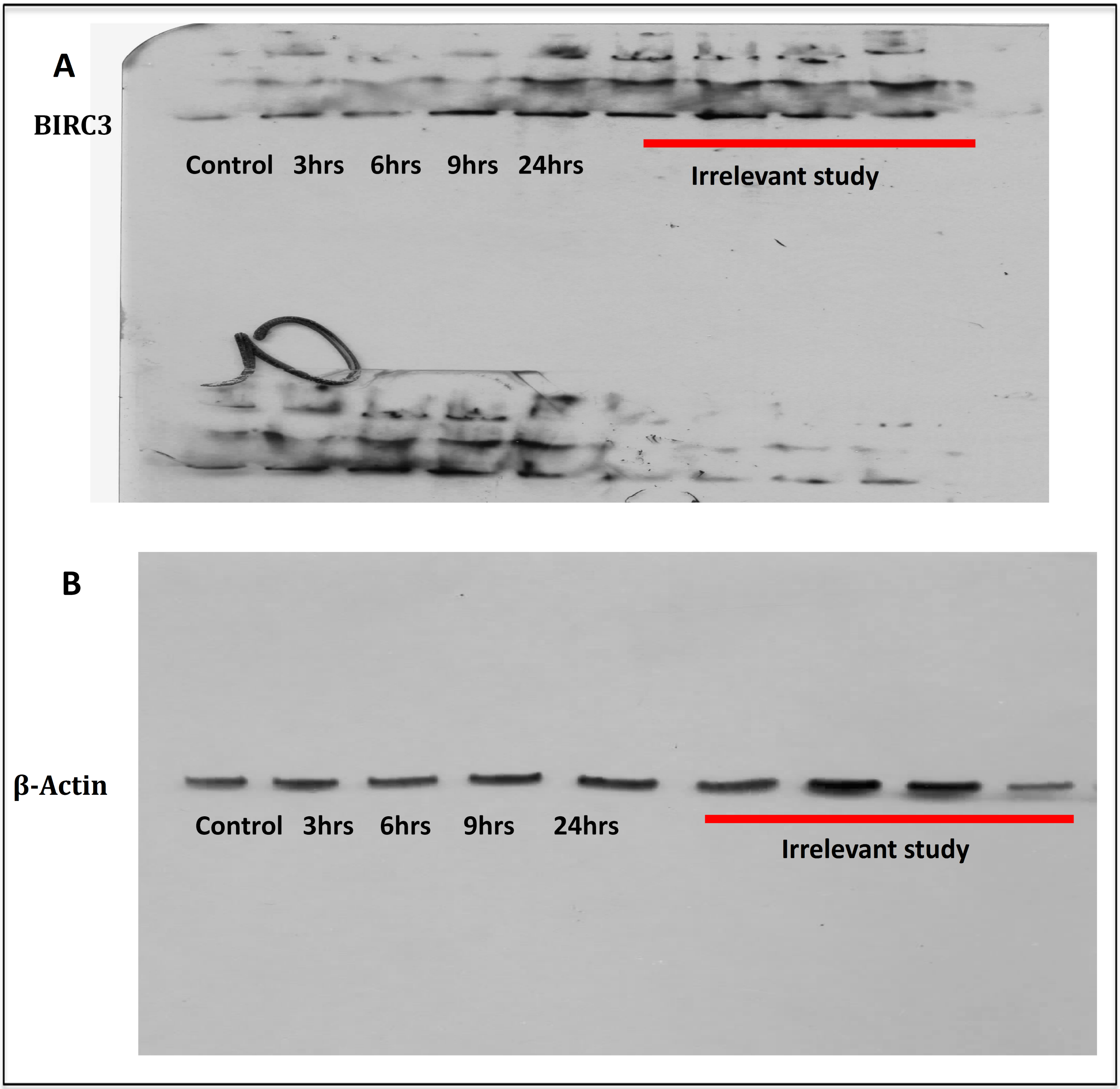

Supplementary Figure 7. Hypoxia induces BIRC3 expression in GBM. U87 cells were cultured under hypoxia conditions (1% O_2) for the indicated intervals and BIRC3 protein levels were determined by western blot. (**A**).BIRC3.(**B**). β-Actin.

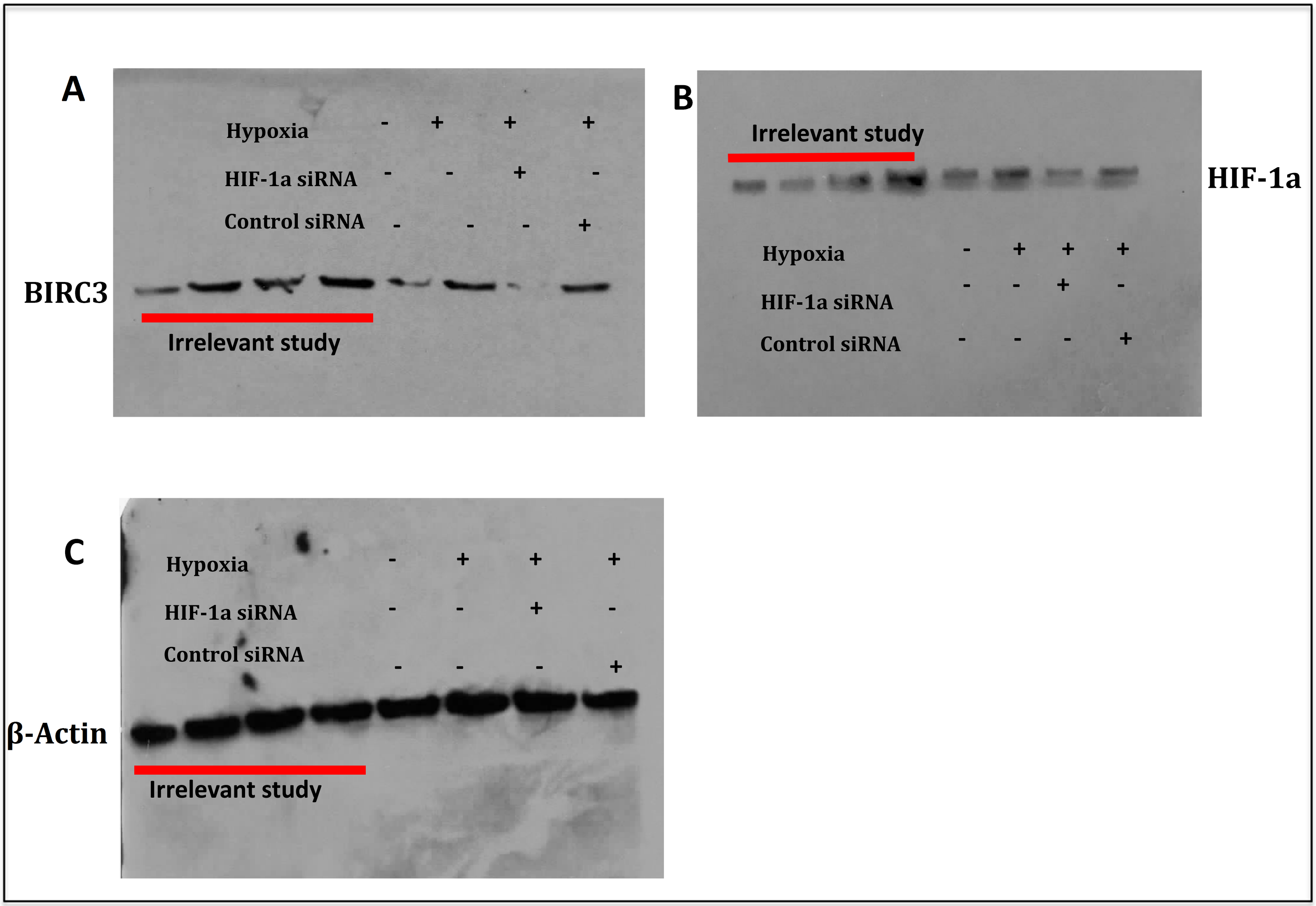

Supplementary Figure 8.Inhibition of HIF-1α blocks hypoxia-induced up-regulation of *BIRC3* expression in GBM. U87 MG GBM cells, or these cells transfected with HIF-1α siRNA for 48 hr, were cultured under hypoxia conditions (1% O_2) for 24 hr. Efficiency of HIF-1α knockdown and effects on BIRC3 gene expression were determined by Western-blot.(**A**) BIRC3. (**B**) HIF-1a.(**C**).β-Actin.

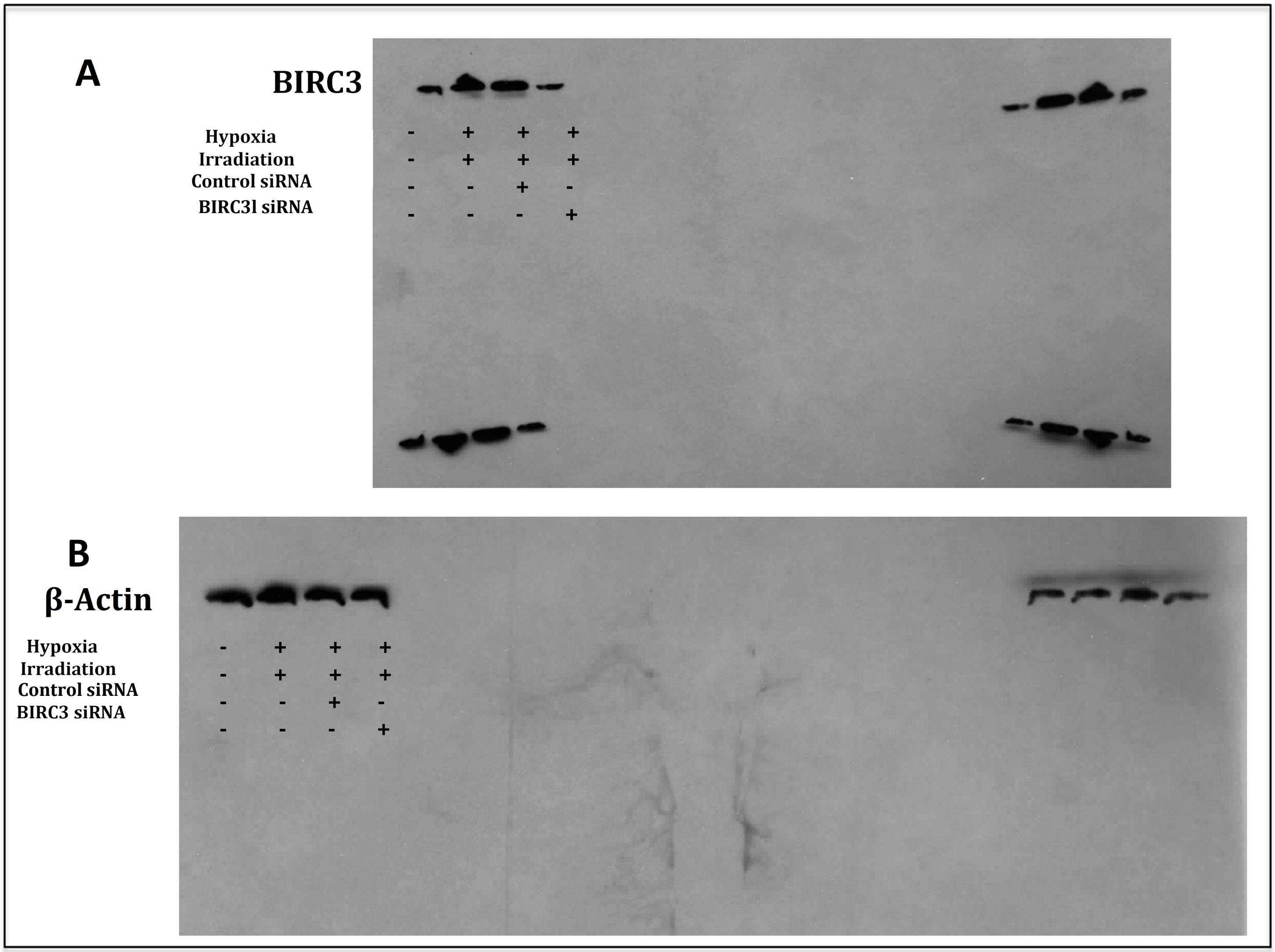

Supplementary Figure 9. BIRC3 silencing impairs hypoxia-induced survival of GBM to radiotherapy (RT). 1×10^4 U87 MG GBM cells were cultured in 96 well plate under hypoxia (1% O_2) condition for 12 hour and irradiated with 4Gy. Cells were returned to hypoxia conditions for another 12 hour and harvested. BIRC3 protein expression were analyzed by Western blot.(**A**) BIRC3. (**B**). β-Actin.
